# Microstructure Evolution and Some Properties of Hard Magnetic FeCr30Co8 Alloy Subjected to Torsion Combined with Tension

**DOI:** 10.3390/ma12183019

**Published:** 2019-09-18

**Authors:** Anna Korneva, Galiya Korznikova, Rishat Kashaev, Boris Straumal

**Affiliations:** 1 Institute of Metallurgy and Materials Science, Polish Academy of Sciences, 30–059 Krakow, Poland; 2 Institute of Problems of Metal Superplasticity, Russian Academy of Science, 450001 Ufa, Russia; korznikova@anrb.ru (G.K.); rishat@kashaev.ru (R.K.); 3 Karlsruhe Institute of Technology (KIT), Institute of Nanotechnology, 76344 Eggenstein-Leopoldshafen, Germany; straumal@mf.mpg.de; 4 Institute of Solid State Physics and Chernogolovka Scientific Center, Russian Academy of Sciences, 142432 Chernogolovka, Russia; 5 National University of Science and Technology «MISiS», 119049 Moscow, Russia

**Keywords:** hard magnetic alloy, severe plastic deformation, gradient microstructure, coercive force, three-point bending test

## Abstract

The hard magnetic alloy FeCr30Co8 alloy was subjected to severe plastic deformation (SPD) by torsion combined with tension in the temperature range of 750 °C to 850 °C. This range of deformation temperatures corresponds to the α solid solution on the Fe–Cr–Co phase diagram. The study of the alloy after SPD by means of X-ray diffraction (XRD) and scanning and transmission electron microscopy techniques showed the formation of a gradient microstructure with fine grain size in the surface layer and precipitation of the hard intermetallic σ-phase. Next, the magnetic and mechanical properties of the deformed alloy after short annealing at 1000 °C and magnetic treatment were studied. A slight decrease in coercive force was found, along with a significant gain in plasticity and strength. The effective deformation temperature was determined to obtain the optimal magnetic and mechanical characteristics of the alloy. This method of deformation can be applied for the improvement of the mechanical properties of some magnets (high-speed rotors) which should have good magnetic properties within their volume while maintaining good mechanical properties on the surface.

## 1. Introduction

The subject of hard magnets with a gradient distribution of properties is of interest to industries seeking materials for high-speed rotors with a strengthened surface layer. This results from the fact that damage to rotors begins in most cases with the appearance of brittle cracks on their surfaces [1]. There are currently various methods of modifying the structure at the surface of materials in order to obtain desired properties. Among these methods, severe plastic deformation (SPD) [2,3], such as surface mechanical grinding treatment via static burnishing the surface with rollers [4] and surface mechanical attrition treatment via dynamic ball peening [5,6,7], is the most popular. These processes, however, suffer from either low processing efficiencies, or limited nanostructured layer thicknesses, or structural inhomogeneity [4,5]. Moreover, the techniques cannot be used in the case of hard magnetic materials in the high coercive state because of their brittleness. In this paper, a unique method of combined load by torsion and tension is proposed as a means of obtaining axially symmetrical permanent magnets with good mechanical properties of the surface and satisfactory magnetic properties within the material. Improved mechanical properties at the surface of the material can be achieved by obtaining an ultra-fine crystalline microstructure with the use of localized severe plastic deformation, while a high level of magnetic properties is ensured by the preservation of a coarse-grain microstructure in the volume of material. 

The Fe–Cr–Co-based alloys are deformable hard magnetic materials of the precipitation-hardening class [8,9]. The alloys of this system combine high magnetic hysteresis properties (residual induction, coercivity, and maximum energy product) with high mechanical properties (strength and ductility) [10]. This explains their wide application in the production of permanent magnets working under conditions of high dynamic loads. Alloys of the Fe–Cr–Co system are susceptible to all kinds of pressure treatment compared to other hard magnetic materials, which allows the production of permanent magnets of various shapes and sizes. A high coercive state of the Fe–Cr–Co system alloys is obtained as the result of magnetic treatment with multistage tempering. The decomposition of the solid α solution into the coherent and ordered precipitates of the α_1_- and α_2_-phases is observed after magnetic treatment [11]. Such a microstructure, in which each precipitate of the α_1_-phase is a single magnetic domain, ensures superior magnetic properties. On the other hand, internal stress fields induced by coherent boundaries between the precipitates of the α_1_- and α_2_-phases cause a reduction of plasticity and strength. The main aim of this work is to study the effect of a gradient microstructure (obtained as a result of plastic deformation by combined tension and torsion) on the improvement of the mechanical properties of the FeCr30Co8 hard magnetic alloy. 

## 2. Materials and Methods

The FeCr30Co8 alloy (59.4 wt % Fe, 29.6 wt % Cr, 7.9 wt % Co, 1.8 wt % Si, 1.1 wt % Ti, 0.2 wt % V) after casting and rolling was subjected to homogenization and water quenching from 1150 °C in order to obtain the α solid solution with a grain size of about 200 µm. Ingots with a diameter of 8 mm were cut by means of spark erosion into cylindrical samples of 44 mm length. Some of these samples were subjected to magnetic treatment to obtain a high coercive state (HCS) without deformation. Other samples underwent SPD by torsion combined with tension. The deformation was carried out on an SNT–10BD (Pisarenko Institute for Problems of Strength of the National Academy of Sciences of Ukraine, Kiyv, Ukraine) complex loading machine, which was modernized at the Institute of Problems of Metal Superplasticity of the Russian Academy of Sciences. This machine allows testing the mechanical properties of materials under complex loading with sequential or simultaneous action of loading elements (torsion combined with tension or compression for solid samples and with additional internal pressure loading for tubular samples) at room temperature or high temperatures [12]. The sample was fixed between the lower and upper anvils of the complex loading machine. Axial deformation and torsion of the sample were carried out by means of the lower anvil. The lower anvil was rotated at a rate of ω = 0.016 rad/s and moved down in a vertical direction at a rate of *V* = 0.0025 mm/s, while the upper anvil was stationary and was connected with the sensors that measure the axial force and torque. The loading between tension and torsion was proportional and the proportionality factor α reached 6.4 rad/mm (α = ω/*R*). It can be seen that torsion made up the main share in the proportional deformation of the sample. The samples were subjected to nine rotations and 20% elongation. The deformation was performed at the temperatures 750 °C, 800 °C, and 850 °C, which correspond to the α-phase area occurrence. The temperatures and rates of deformation corresponded to the superplasticity conditions of the examined alloy [13]. The total degrees of cumulated plastic deformation were calculated at distances of 3.5 and 2 mm from the center of the deformed samples using Equation (1):

(1) $$e_{e}=ln\left(1+\frac{\Delta L}{L}\right)\sqrt{1+{\left(\frac{\propto R}{\sqrt{3}}\right)}^{2}}$$

where *R* (mm) is the distance to the deformed sample’s rotation axis; Δ*L* (mm) is the tensile gain; and *L* (mm) is the sample length after deformation. 

**Figure 1:**
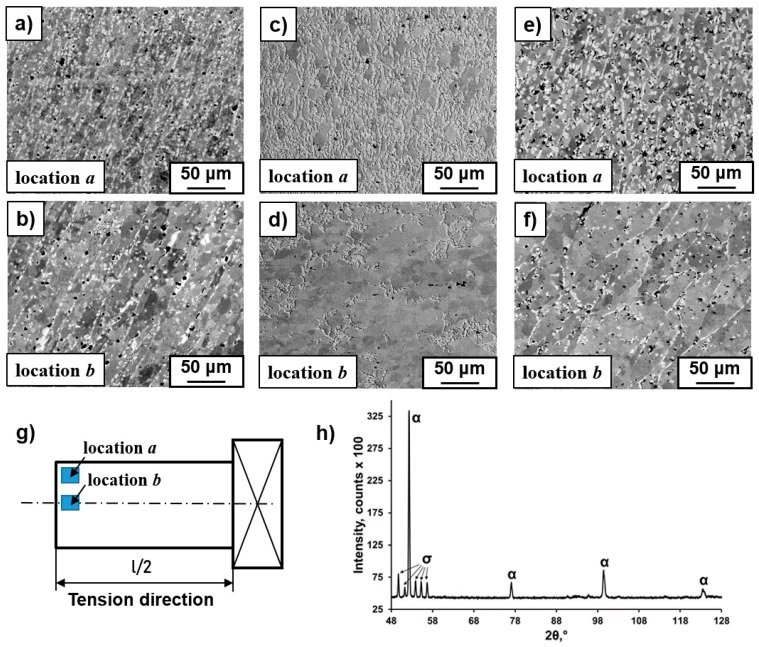
SEM images of the microstructure at the surface (**a**,**c**,**e**) and in the volume (**b**,**d**,**f**) of the samples deformed at 750 (**a**,**b**), 800 (**c**,**d**), and 850 °C (**e**,**f**) after nine rotations of torsion and 20% tension. The σ-phase has bright contrast in comparison with the dark α-phase in the SEM images obtained in back-scattered electron mode (BSE). The scheme of microstructure observations in SEM (**g**). XRD pattern of the alloy deformed at 800 °C (**h**).

The microstructure observations were performed by means of an XL 30 ESEM scanning electron microscope (SEM), Philips (Hillsboro, OR, USA), with a LINK ISIS energy-dispersive X-ray spectrometer (EDS) produced by Oxford Instrument (Hillsboro, OR, USA). The SEM microstructure observations were performed at the distance of a half-length of the samples at their longitudinal section (Figure 1g). The SEM images were taken using backscattered electron signal (BSE) mode in order to obtain the composition contrast between different phases. The specimens for SEM observations were ground with SiC grinding paper and sequentially polished with 6 µm, 3 µm, and 1 µm diamond pastes. Next, the samples were electrolytically polished in an electrolyte composed of 10 ml of hydrochloric acid and 1000 ml of butanol. Chemical etching was carried out in a reagent composed of 5 drops of glycerol, 15 ml of hydrochloric acid, 15 ml of acetic acid, and 10 ml of nitric acid HNO_3_. The details of the microstructure in nanoscale were revealed using a TECNAI G2 FEG super TWIN (200 kV) transmission electron microscope (TEM) equipped with an energy dispersive X-ray (EDS) spectrometer manufactured by EDAX TECNAI (Mahwah, NJ; USA). Thin foils of the alloy for TEM observation (TECNAI, Hillsboro, OR, USA) were prepared by a twin-jet polishing technique using the electrolyte composed of 10 ml of hydrochloric acid and 1000 ml of butanol.

The magnetic treatment was carried out according to Russian Standard number 24897-91 [14] for solid magnetic deformed materials: heating at 620–650 °C in a magnetic field with intensity of at least 100 kA m^−1^ for 1–2 h and tempering at 610 °C for 2 h, at 580 °C for 3 h, at 560 °C for 4 h, and at 540 °C for 6 h. The magnetic field direction was parallel to the tension direction. Magnetic measurements were carried out on cuboidal samples with dimensions 3 × 1.7 × 15 mm by means of a Permagraph C–300 magnetometer (Magnet-Physik Dr. Steingroever GmbH, Germany), while the external magnetic field was parallel to the tension direction. The scheme of sample cutting for magnetic treatment is presented in Figure 4a. After magnetic treatment the mechanical properties were examined by the three-point bending technique at room temperature on an “Instron 5982” machine. The stress tension was calculated using Equation (2) [15]:

(2) $$\sigma =\frac{3\cdot P\cdot L}{2\cdot b\cdot h^{2}}$$

where *P* (N) is force; *L* (mm) is the distance between support points; and *b* (mm) and *h* (mm) are the width and height of the sample.

X-ray diffraction analysis (XRD) was performed with a PW-1710 (Philips, Eindhoven, Netherlands) diffractometer using Co K_α_ radiation. 

## 3. Results and Discussion

XRD analysis and SEM observations of the initial state of the FeCr30Co8 alloy showed only the reflections of an α solid solution with a grain size of about 200 µm [16]. After plastic deformation, an axially symmetrical gradient microstructure was obtained with minimal grain size in the surface layer. SEM images of the microstructure at the surface and in the volume of the deformed samples are shown in Figure 1. The total degrees of deformation reached 2.0 and 1.1, measured at distances of 3.5 mm and 2 mm from the center of the deformed samples, respectively. It can be seen that the degree of deformation close to the surface is higher than that close to the center of the samples, which indicates greater deformation at the surface, in particular by the action of the highest torsion moment. The thicknesses of the surface layer with the fine-grained microstructure were about 2.0 mm, 3.0 mm, and 2.5 mm for the samples deformed at 750 °C, 800 °C, and 850 °C, respectively. The microstructural investigations performed by EBSD/SEM and TEM analysis of these deformed samples were published in [16]. These studies showed that the highest grain refinement of the microstructure was observed at 800 °C; only at this temperature were the globular α-phase grains (sizes of about 3 µm) at the surface layer observed, while at 750 °C and 850 °C they were elongated (up to 35 µm) in the direction perpendicular to the tension axis. 

**Figure 2:**
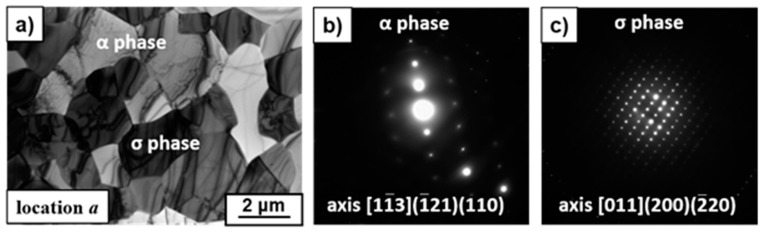
TEM image of the microstructure (**a**) and diffraction patterns of the α-phase (**b**) and the σ-phase (**c**), observed at the surface of the sample deformed at 800 °C.

**Figure 3:**
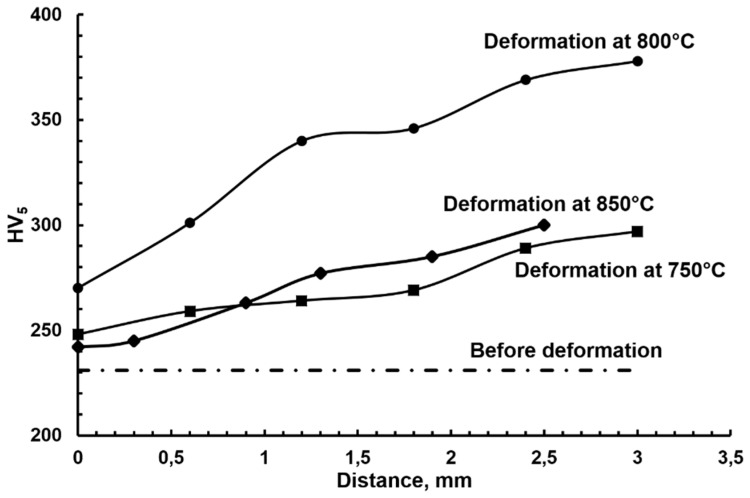
Graphs of hardness measured along the line perpendicular to the tension direction. The distances 0 mm and 3 mm correspond to Locations *b* and *a*, respectively, in the deformed samples.

XRD (Figure 1g) and EDS/SEM analysis revealed the appearance of the intermetallic σ-phase (Fe–Cr) with chromium content up to 40 wt % and grain sizes of about 5 μm (bright color in Figure 1a–f). The precipitation of the σ-phase had a diffusion character because it precipitated generally in the grain boundaries of the α-phase (Figure 2) with increased energy, which facilitates diffusion processes. According to the EBSD/SEM results, the amounts of the σ-phase in the surface layer were 12%, 43%, and 22% and were 2%, 12%, and 10% in the volume for the samples deformed at 750 °C, 800 °C, and 850 °C, respectively [16]. It was found previously that the σ-phase for the alloys of the Fe–Cr–Co system can be observed after annealing at temperatures ranging 560 °C to 800 °C; however, the process of its precipitation requires several days of annealing [17]. Based on the fact that the maximum precipitation of the σ-phase was recorded in the places of intensive deformation (i.e., in the surface layer), it seemed that deformation stimulated the σ-phase precipitation due to the activation of diffusion processes. It is probable that a stronger thermal activation of diffusion processes at the deformation temperature of 800 °C is the reason for the increase of the σ-phase amount in comparison to the amount observed in the sample deformed at 750 °C. The decrease in the fraction of precipitates at 850 °C was due to the fact that the σ-phase was no longer stable at this temperature. Since the highest precipitation of the σ-phase and refinement of the microstructure took place in the same sample (deformed at 800 °C), it was suggested that σ-phase precipitation promoted microstructure refinement [16]. A considerable increase of hardness at the surface of the deformed samples was observed also due to the strong grain refinement of the microstructure and the presence of the hard σ-phase in the material [16]. For example, the hardness of the initial state was about 230 HV_5_. After deformation at 800 °C the hardness increased to 270 HV_5_ (Location *b*) and 380 HV_5_ (Location *a*) (Figure 3). Therefore, this method of plastic deformation can be applied for the surface hardening of magnets dedicated for work in friction conditions, since the hard σ-phase protects their surface from abrasion.

On the other hand, the precipitation of nonmagnetic σ-phase is disadvantageous due to decreased magnetic properties. Therefore, before magnetic treatment, the deformed samples were subjected to annealing at 1000 °C for 10 min, followed by fast cooling in order to dissolve the σ-phase. The samples were then magnetically treated in order to achieve an HCS due to decomposition of the solid α-solution into the isomorphous α_1_- and α_2_-phases. The formation of a such structure, in which each precipitate of the α_1_-phase is a single magnetic domain, provides superior magnetic properties [11].

The microstructure of samples after magnetic treatment disclosed α-phase grain growth and the preservation of a gradient structure. The sizes of the α-phase grains were about 56 µm, 60 µm, and 87 µm in the top layer and 87 µm, 76 µm, and 119 µm in the volume of the samples pre-deformed at 750 °C, 800 °C, and 850 °C, respectively. 

**Figure 4:**
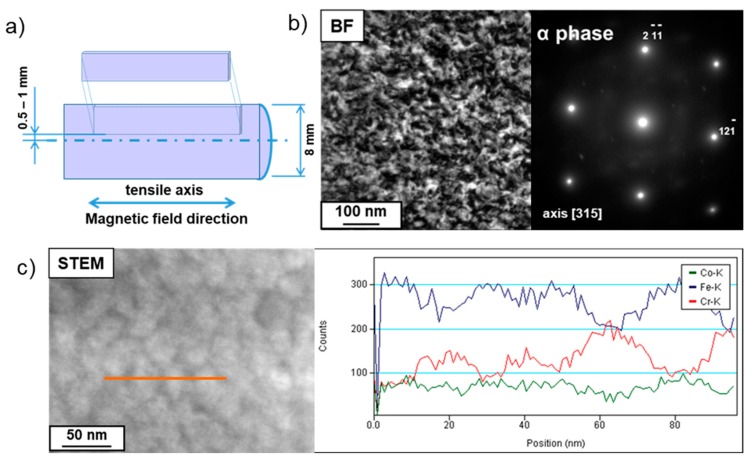
Scheme of the sample cutting for magnetic treatment (**a**). Bright field image and diffraction of the microstructure obtained from the surface layer of the sample deformed at 800 °C after magnetic treatment (**b**), TEM. STEM image of the same microstructure and distribution of Co, Fe, and Cr along the line of analysis in image (**c**).

TEM examinations of the microstructure after magnetic treatment confirmed the decomposition of the α-phase into the magnetic α_1_-phase enriched in Fe (up to 84 atom %) and the paramagnetic α_2_-phase rich in Cr (up to 36 atom %). The diffraction contrast was responsible for difficulties in recognizing both phases in the bright field image (Figure 4a), but in the scanning microscope (STEM) mode the problem was overcome, and so the α_1_ precipitates with sizes of about 30 nm in the α_2_-matrix could be seen (Figure 4b). The distribution of chemical elements along the line of analysis showed the sinusoid-like character of the Fe and Cr changes. The Co level was practically unchanged for both phases.

**Figure 5:**
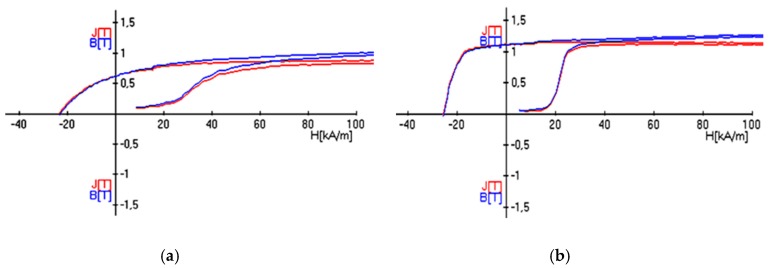

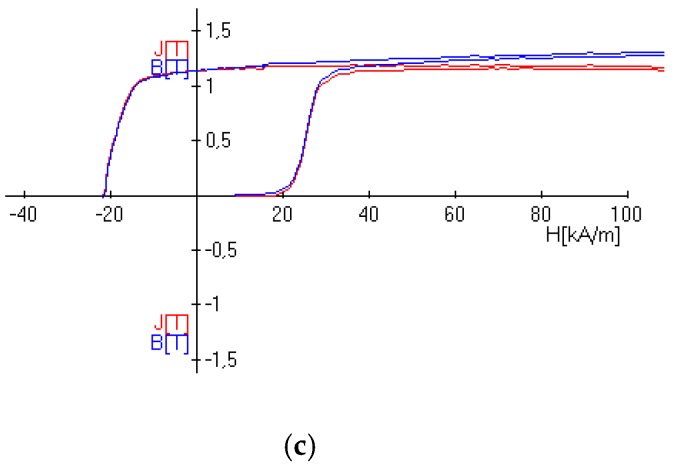
Magnetization curves of the samples after deformation by tension and torsion at 750 °C (**a**), 800 °C (**b**), and 850 °C (**c**) and magnetic treatment.

The curves of hysteresis loops of the new samples previously deformed at 800 °C and 850 °C were nearly perpendicular in shape (Figure 5b,c), which indicated high parameters of residual induction B_r_ (Table 1) and magnetic product (BH)_max_. The sample pre-deformed at 750 °C had a hysteresis loop of rather flat shape, which was probably connected with distinct differentiation of the dimensions of the α grains. The coercive force H_c_ of all samples was 9% to 22% less than that in the HCS without deformation (Table 1). The decrease of the coercive force was connected with the refinement of the microstructure; the lower the grain size, the higher the number of high-angle grain boundaries, so the volume fraction of the material taking part in the decomposition of the α-phase into the α_1_- and α_2_-phases was lower.

**Figure 6:**
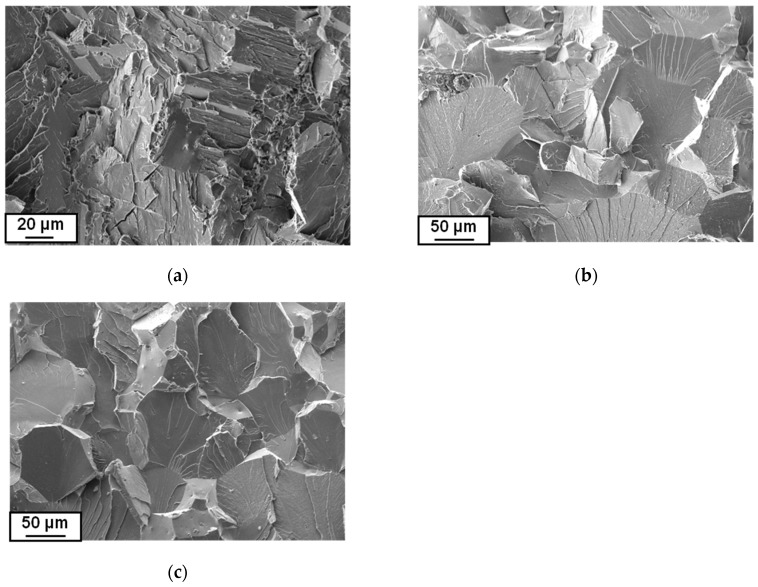
Fractures of samples after the three-point bending test, pre-deformed at 750 °C (**a**), 800 °C (**b**), and 850 °C (**c**).

Measurements of the mechanical properties with the use of the three-point bending method after magnetic treatment were performed at ambient temperature. Fracture analysis after the bending test revealed a brittle trans-crystalline type of damage for the samples pre-deformed at 800 °C and 850 °C (the propagation of cracks proceeded mainly along crystallographic planes, Figure 6). The fracture of the sample which was pre-deformed at 750 °C showed elements of ductile cracking in the form of characteristic cavities, which was probably bound with the differentiation of grain dimensions. The brittle fracture occurring at higher temperature deformations could be explained by the presence of bigger grains in which obstacles for crack propagation did not exist. Apart from that, decomposition of the α-phase in the material of large grain size during magnetic treatment took place to a great extent. During the decomposition, internal strains appeared, coming from coherent boundaries between the α_1_- and α_2_-phases; in such a material, strains were more frequently observed and were the reason for more brittle cracking. 

The maximum stresses, which correspond to the stage of plastic flow on stress–displacement curves from the three-point bending test, reached 1071 MPa, 877 MPa, and 742 MPa for the samples pre-deformed at 750 °C, 800 °C, and 850 °C, respectively. These values exceeded 2 to 2.7 times the strength of the alloy in the HCS without deformation, which was destroyed in the range of elastic strain (Table 1). As the temperatures of pre-deformation increased, the stresses decreased, which may be related to the thermal activation of diffusion processes. It should be noted that the material in the HCS after casting revealed no plasticity, while the material deformed by tension and torsion had increased plasticity. The maximum size of deflection was observed in the sample deformed at 750 °C and reached about 0.3 mm (Table 1). This resulted from the greater grain refinement and homogeneity of the microstructure in respect of the chemical composition. It should be noted that close values of mechanical properties were obtained for the FeCr25Co15 alloy, measured in the three-point bending test [18]. This alloy had previously been subjected to complex loading by axial compression and torsion in the temperature range of 700 °C to 850 °C and to magnetic treatment.

It is a challenge to produce materials with both high strength and high ductility. It was shown recently that materials with a gradient structure can exhibit a combination of these properties [19,20]. For example, the authors of [19] showed that the torsion deformation of Cu rods resulted in the formation of gradient lamellar dislocation substructures (LDS) along the radial direction as the main defects. High-density LDS with fine spacing can retain strain hardening capability, which resulted in a good combination of high tensile strength and good ductility in the torsional deformed sample with a large number of revolutions. 

## 4. Conclusions

A method of plastic deformation with torsion and tension at elevated temperatures applied to FeCr30Co8 alloy proved to be an efficient tool to form a gradient microstructure with fine grains in the surface layer. It also enabled an increase of the strength and plasticity of the material in the high coercive state.

The effective temperature of plastic deformation for FeCr30Co8 alloy prior to magnetic treatment was 800 °C, because at that temperature, the alloy gained maximum structure refinement, maximum precipitation of the σ-phase, and a significant increase in hardness. However, the best strength properties and plasticity values after magnetic treatment were achieved in the sample deformed at 750 °C, which preserved the same level of coercive force characteristics for samples deformed at higher temperatures.

## Figures and Tables

**Table 1:** Magnetic properties (coercive force H_c_, residual induction B_r_, saturation flux density B_s_, magnetic product (BH)_max_) and mechanical properties (maximum stress σ, size of deflection ε) of the deformed alloy after magnetic treatment and three-point bending test.

State	Magnetic Properties				Mechanical Properties	
	H_c_, A/_M_	B_r_, T	B_s_, T	(BH)_max_, kJ/m^3^	σ, MPa	ε, mm
750 °C	23.4	0.61	1.01	5.1	1071	0.31
800 °C	25.5	1.11	1.73	16.7	877	0.16
850 °C	21.8	1.13	1.79	14.3	742	0.17
High Coercive State without deformation	28.0	1.15	1.8	18	392	0.0
